# Microbiota Targeted Interventions of Probiotic *Lactobacillus* as an Anti-Ageing Approach: A Review

**DOI:** 10.3390/antiox10121930

**Published:** 2021-11-30

**Authors:** Muhammad Ishaq, Ashiq Khan, Ali Sher Bacha, Tariq Shah, Anum Hanif, Anum Ali Ahmad, Wencan Ke, Fuhou Li, Ahmad Ud Din, Zitong Ding, Xusheng Guo

**Affiliations:** 1 School of Life Sciences, Probiotics and Biological Feed Research Centre, Lanzhou University, Lanzhou 730000, China; ishaq2017@lzu.edu.cn (M.I.); ashiq2017@lzu.edu.cn (A.K.); ali2018@lzu.edu.cn (A.S.B.); tariq.shah00@gmail.com (T.S.); anum17@lzu.edu.cn (A.H.); anum2017@lzu.edu.cn (A.A.A.); kewc12@lzu.edu.cn (W.K.); lifh17@lzu.edu.cn (F.L.); 2 Department of Microbiology, Balochistan University of Information Technology Engineering & Management Sciences, Quetta 87300, Pakistan; 3 Drug Discovery Research Center, Southwest Medical University, Luzhou 646000, China; ahmadnwa@swmu.edu.cn

**Keywords:** probiotic *Lactobacillus*, ageing, gut microbiota, oxidative stress, elderly

## Abstract

With the implementation of modern scientific protocols, the average human lifespan has significantly improved, but age-related problems remain a challenge. With the advent of ageing, there are alterations in gut microbiota and gut barrier functions, weak immune responses, increased oxidative stress, and other age-related disorders. This review has highlighted and discussed the current understanding on the significance of gut microbiota dysbiosis and ageing and its inherent effects against age-related oxidative stress as well as on the gut health and gut-brain axis. Further, we have discussed the key mechanism of action of *Lactobacillus* strains in the longevity of life, alleviating gut dysbiosis, and improving oxidative stress and inflammation to provide an outline of the role of *Lactobacillus* strains in restoration of gut microbiota dysbiosis and alleviating certain conditions during ageing. Microbiota-targeted interventions of some characterized strains of probiotic *Lactobacillus* for the restoration of gut microbial community are considered as a potential approach to improve several neurological conditions. However, very limited human studies are available on this alarmed issue and recommend further studies to identify the unique *Lactobacillus* strains with potential anti-ageing properties and to discover its novel core microbiome-association, which will help to increase the therapeutic potential of probiotic *Lactobacillus* strains to ageing.

## 1. Introduction

Ageing is a complex process characterized by decline in physiological function caused by continuous deteriorations and changes at cellular and tissue level. It is a predestined physiological phenomenon attributed to the low functional capability of an organ’s functioning. Researchers have focused on its possible mechanism. Still, these studies are complex because the process and pace of ageing is greatly varied among individuals [1]. Inside cells, ageing can be evident as the loss of function or unrestrained propagation. Both microscopic and macroscopic changes occur in tissues with indefinite composition and elevated cross linking in the extracellular matrix, which causes stiffness and the loss of mechanical toughness [2]. Constant cell damage is considered as the ultimate reason for tissue failure; hence, ageing is an important risk factor for several diseases. To understand the actual reasons for our mortality, studies have been focused on ageing to sustain the quality of life in the ageing population globally. However, ageing studies are complex because ageing is influenced by genetic and environmental factors throughout life, which also shows great discrepancy among the subjects [3].

The age-related changes in gut microbiota favor the growth of pathogens and gut-associated diseases. Ageing also affects the composition of beneficial microbes in gut, where their abundance and diversity gets lower [4]. There exists a link between dysbiosis and age-associated metabolic disorders during ageing. The elderly has a low diversity of gut microbiota, specifically linked to metabolic disorders (as evident from animal model studies), which further leads to higher inflammation and compromised immune system. Therefore, the maintenance of a stable gut microbiota is crucial for healthier ageing. One of the key reasons for ageing is the decline in metabolism due to continuous exposure to oxidative stress, which further causes mitochondrial dysfunction [5]. So far, most of the previous studies have focused on feeding live probiotic strains to the animals in ageing experiments. However, the use of heat-killed probiotics has several benefits, including safety and long-lasting effects. Heat-killed probiotics confer different biological effects, such as stimulating the intestinal immune responses and anti-inflammatory effects. A recent study showed that the intra-gastric feeding of heat-killed *Lactobacillus paracasei* PS23 to aging mice can improve age-related muscle atrophy. Ghrelin is an essential gut hormone with pleiotropic effects that controls hunger, meal initiation, and nutrient sensing. It can also indirectly improve muscle mass by stimulating the IGF1 pathway in mice with cachexia. The heat-killed PS23 can help maintain the levels of ghrelin in ageing mice; hence, it can improve the muscle function during ageing [6].

Probiotics are defined as live microorganisms that confer a health benefit on the host when administered adequately. The most common probiotic strains are associated with the genera *Lactobacillus* and *Bifidobacterium*. Recent advancements have witnessed significant achievements in using *Lactobacillus* during animal studies in ageing models [7]. Their key benefits on the host health include improving the barrier function, immunomodulation, and the production of neurotransmitters. Additionally, they can positively influence the host gut microbiota and cellular components of gut-brain axis. 

This review has highlighted the advances in using *Lactobacillus* as therapeutic interventions to improve ageing via microbiota-targeted approach. The initial focus is the effectiveness of different *Lactobacillus* strains against age-related oxidative stress, including anti-immunosenescence effects of *Lactobacillus*. Other sections include microbiota targeted effects of *Lactobacillus* on skin and brain ageing. 

## 2. Ageing-Related Gut Microbiota Dysbiosis and the Role of *Lactobacillus*

Infants get their microbiome either at the time of or before birth, and they may be exposed to bacteria via the birth canal or in case of contact with their maternal skin (Caesarian born babies) [8]. In a newborn baby, the gut colonization begins with facultative anaerobic bacteria, like enterobacteria and streptococci, which further continues with anaerobes, like *Bifidobacterium*, *Clostridium,* and *Bacteroides* [9]. Microbial composition in the gut of a newborn baby undergoes significant changes due to different environmental and genetic factors and eventually develops a commensal intestinal microbiome. The gut of every adult human carries about 10^14^ bacteria, approximately 10-fold the number of cells that make up the human body [10]. This ecosystem is home to a minimum of 400–500 bacterial species divided into various strains that highlight its huge intricacy [11].

The most prominent bacterial species inside the gut of a healthy human mostly consist of *Bacteroidetes* and *Firmicutes* [12]. *Proteobacteria*, *Actinobacteria*, *Fusobacteria*, *Cyanobacteria,* and *Verrucomicrobia* are less abundant phyla [13]. Furthermore, different sites are inhabited by various bacterial groups. Bacilli (a class of *Firmicutes* and *Actinobacteria*) are more abundant in the small intestine, while the families of *Firmicutes* (*Bacteroidetes* and *Lachnospiraceae*) are more abundant in the colon region [14]. Additionally, there are significant differences in the intestinal lumen and mucosal layer’s microbiota composition and the microbiota present near the epithelium [15]. 

The age-related changes in the composition of gut microbiota include a decline in diversity, decreased population of saccharolytic bacteria and increased proteolytic bacteria [16], a decline in the abundance of core (dominant) species and higher abundance of subdominant species [17], an increase in the number of certain proteobacteria, and a reduction of *Bifidobacteria* and *Firmicutes* to *Bacteroides* ratio [18,19]. For instance, in the vaginally delivered breast-fed infants, the numbers of *Bifidobacteria* decreases throughout the course of life from 90% of the total colon microbiota following birth to less than 5% in the colon of adults, and these numbers were even less in the elderly [20]. The major changes in gut microbiota take place through the shift from adulthood to old age. Elderly people (older than 65) are characterized by a decline in the microbial diversity, and a greater inter-individual differences in microbiota diversity have been observed compared to adults [21], with less numbers of *Bifidobacteria*, *Firmicutes*, *Faecalibacterium prausnitzii*, *Clostridium* cluster XIV, *Blautia coccoides, Eubacteriumm rectal,* and greater incidence of *Bacteroides* and *Enterobacteriaceae* [19]. However, it is worth noting that the data on age-related alterations in microbiota composition are different form population to population

*Lactobacillus* colonizes the gut and inhibits gut pathogens through competitive exclusion, which includes occupying the adhesion sites, consuming nutrients, and producing antimicrobials [15]. As the role of probiotic bacteria in the composition of gut microbiota and gut diseases has been studied, few studies have focused on the effects of probiotic bacteria concerning the diversity of gut microbiota. Probiotics are regularly residing the gastrointestinal tract, which can regulate the microbiota composition of the intestinal tract and restrict the growth of pathogenic strains. *Lactobacillus* strains can potentially reshape the gut microbial composition by restoring the diversity and inhibiting the growth of pathogens. Supplementation of *Lactobacillus acidophilus* and *Lactobacillus casei* increased the abundance of lactic acid bacteria with a corresponding decrease in fecal coliforms and anaerobes [22]. In a study, the gut microbiota composition was restored by the supplementation of *Lactobacillus helveticus* KLDS1.8701 up to the level of the control group, and butyrate production was increased with a corresponding decrease in endotoxins production [23]. The administration of the strain *L. paracasei* J1us66 could attenuate the structure of intestinal microflora by enhancing the population of gram-positive bacteria, like *Firmicutes,* and inhibiting gram-negative microbes, like *Bacteroidetes*, *Proteobacteria,* and *Fusobacteria* [24]. Li et al. reported that a mixture of probiotic bacteria, including *Lactobacillus,* enhanced the abundance of beneficial bacteria, such as *Prevotella* and *Oscillibactor,* which are known for the production of anti-inflammatory metabolites [25]. *Lactobacillus johnsonnii* BS15 altered the *Firmicutes*/*Bacteroidetes* in the gut [26]. The supplementation of two *Lactobacillus* strains (*L. curvatus* HY7601 and *L. plantarum* KY1032) enhanced gut microbiota in HFD-fed mice [27]. Along with enhancement of intestinal endotoxemia, a mixture of probiotics (*Bifidobacterium infantis, Lactobacillus acidopilus,* and *Bacillus cereus*) resulted in the increased abundance of these anaerobic microbes and decreased the levels of *Escherichia coli* and *Enterococcus* species in the fecal samples of high-fat and high-sugar fed rats [28].

Strains of *Lactobacillus* can effectively improve digestion, sustain intestinal health in situations such as atopic dermatitis and gastroenterological disorders, and reverse the dysbiosis of gut microbiota to reestablish the gut mucosal homeostasis [29]. In-vitro experiments to define the molecular mechanism of probiotics have countable effects for strain-specific and molecule specific advantages to the host, such as improving the anti-inflammatory cytokine profiles and stabilizing tight epithelial junctions. Well-distinguished stimulator molecules produced by probiotic bacteria contain cell surface proteins, lipoteichoic acid, muropeptides, peptidoglycan-derived, pilus-type morphology, and exopolysaccharides [30]. Alternatively, performing such in-vivo experiments to explain probiotics’ mechanism is complicated and challenging due to several factors, such as relation of the host organism with the microbiota nearby gastrointestinal tract. *Lactobacillus rhamnosus* GG ATCC 53103 (LGG) has health benefits during clinical trials in various cohort studies. It is considered to act through (a) competitive colonization by using the mucus-binding pili, (b) predicted activity of bacteriocins confirmed through bacteriocin-like genomic architecture, and (c) soluble effectors-activating proteins that generate anti-inflammatory cytokines and trigger mitogen-activated protein kinases (MAPKs) [31,32]. Although LGG can form strong biofilm during in-vitro experimental setup, during in-vivo experiments, their role is limited to only small biological slots and the GIT of humans with limited biofilm formation [33]. The use of a phylogenetic microarray (HIT Chip) to evaluate structure and diversity of fecal microbiota of a healthy adult Finish cohort detected no remarkable changes associated with LGG consumption [34]. The main challenge to analyze the effects of *Lactobacilli* on the surviving gut microbiota is the potential to determine both the structural and functional mechanisms on a communitywide level. Nowadays, techniques and sequencing technologies are becoming available for the sampling of active gut microbiota.

Using new technologies to probe the metatranscriptomic data, Emiley et al. analyzed the structural and functional behaviors (gene expression) of the gut microbial community during the utilization of a probiotic bacteria *L. rhamnosus* GG ATCC 53103 (LGG) from the analysis of 12 healthy elders. The gut microbiota of elders furnishes characteristic pattern to explain the exact situation of the provisional healthy ageing microbiota and elaborate the dynamics and stability of the community. They noticed no change in composition of the community as a result of probiotics consumption, but there were clear changes in the community-wide transcriptomic data [35]. Thus, the above evidence shows that the supplementation of various *Lactobacillus* strains can greatly influence and restore the ageing gut microbiota. They can act through different ways, such as occupying the adhesion sites of the intestinal wall, competing for the nutrients, and producing lactic acids to maintain low intestinal pH, which can inhibit the growth of certain pathogenic microbes and produce certain antimicrobial metabolites like bacteriocins. Different animal model studies suggest that the consumption of different strains can restore the gut microbiota diversity to some extent (Table 1).

Age-related degenerative changes are usually considered to be associated with microbial imbalance in the gut. Before the administration of *L. plantarum* CCFM10, the composition of microbiota in D-gal induced ageing mice was characterized by higher *Firmicutes*/*Bacteroidetes* ratio. At genus level, the relative abundance of *Lactobacillus* was decreased while the proportion of a genus of clastridiales was increased. After the administration of L. plantarum CCFM10, the microbiota composition was in line with the control group both at genus and phylum levels, reflecting the potential of this probiotic strain to restore the microbiota. This study shows that the protective effects of this strain on the resident microbiota could be it resistance to oxidative stress [36]. In a study, the administration of heat-killed *L. paracasei* KW3110 for six months significantly reversed the age-related alterations in gut microbiota composition in aged mice. The population of *Bifidobacterium* was significantly enhanced though this bacteria is not usually detected in the elderly. The ratio of *Firmicutes* to *Bacteroidetes* was increased in the control group as compared to the mice fed with the strain *L. paracasei* KW3110. Higher *Firmicutes*/*Bacteroidetes* ratio is associated with intestinal inflammation. The intake of this strain might resist intestinal inflammation by balancing the *Firmicutes*/*Bacteroidetes* ratio [37]. A lower *Bacteroidetes*/*Firmicutes* ratio is considered to be associated with abnormal conditions, such as hypertension and obesity. The long-term intake of LB81 yogurt supplemented with *L. delbrueckii* *sub sp. Bulgaricus* 2038 has been associated with higher *Bacteroidetes/Firmicutes* ratio, which is an indication that this strain can prevent the diseases associated with dysbiosis. The abundance of proteobacteria was also increased [38]. The administration of *L. acidophilus* DDS-1 increased the relative proportion of *Firmicutes,* while *Bacteroidetes* were decreased. The abundance of beneficial bacteria, such as *Verrucomicrobia* and *Akkermansia muciniphila,* was significantly enhanced, while the levels of opportunistic bacteria, such as *Proteobacteria,* were decreased in the ageing group after the probiotic treatment. Treatment with the strain *L. plantarum* TWK10 significantly reversed the age-related dysbiosis in the ageing mice. The results indicated that *Firmicutes*/*Bacteroidetes* ratio was decreased in the aged mice after treatment with the probiotic strain. The phylum *Proteobacteria* is comprised of a range of gram-negative bacteria (pathogenic), such as *Enterobacteriaceae, Pseudomonadaceae, Vibrionaceae*, and *Versiniaceae*. Among these, *Enterobacteriaceae* is a core microbial group representing over 30 genera and 130 species. These *Enterobacteria* also include potentially pathogenic microbes having specific effects on the host mucosal immune system and are the major cause of infection when the host immune system fails to resist the infection with age. Treatment with *L. plantarum* TWK10 significantly reduced the abundance of *Enterobacteriaceae* in the aged mice [39].

**Table 1:** List of different studies indicating the impact of *Lactobacillus* strains on the restoration of resident gut microbiota in different animal models during ageing.

Microbiota Diversity of Experimental Animals during Age-Related Conditions at Different Levels before Probiotic Supplementation			Strains of the Genus *Lactobacillus* Supplemented in the Studies	Microbiota Diversity after the Probiotic Supplementation		Ref:
Phyla	Increase	Decrease		Increase	Decrease	
*Firmicutes*	√	√	*L. plantarum* CCFM10, *L. delbrueckii* *subsp. Bulgaricus* 2038, *L. acidophilus* DDS-1, *L. helveticus OFS 1515 and L. fermentumDR9,* *L. casei LC122*	√		[36,38,40,41,42]
	[36,37,39]	[38,40,41,42]				
			*L. paracasei KW3110, L. plantarum* TWK10		√	[37,39]
*Bacteroidetes*	√	√	*L. delbrueckiisubsp. Bulgaricus* 2038, *L. casei* *LC122, L. plantarum* TWK10	√		[38,39,42]
	[38,41]	[36,37,39,40,42]	*L. plantarum* CCFM10, *L. acidophilus* DDS-1, *L. paracasei KW3110,* *L. helveticusOFS 1515 and L. fermentumDR9*		√	[36,37,40,41]
F/B ratio	√	√	*L*. *plantarum* CCFM10, *L. acidophilus* DDS-1	√		[36,40]
	[36,37,39,42]	[38,40,41]				
			*L. paracasei KW3110, L. delbrueckiisubsp. Bulgaricus* 2038, *L. helveticus OFS 1515 and L. fermentum DR9,* *Lactobacillus casei LC122, L. plantarum* TWK10		√	[37,38,39,41,42]
TM7	√	√	*L. delbrueckiisubsp. Bulgaricus* 2038,	√		[38]
	[36]	[38]				
			*L*. *plantarum* CCFM10		√	[36]
*Proteobacteria*	√	√	*L. plantarum* CCFM10, *L. delbrueckii* *subsp. Bulgaricus* 2038, *L. paracasei KW3110,* *L. casei LC122*	√		[36,37,38,42]
	[36,39,40]	[37,42]				
			*L. acidophilus* DDS-1, *L. plantarum* TWK10		√	[39,40]
*Deferrebacteres*		√	*L. paracasei KW3110*	√		[37]
	[40]	[37]				
			*L. acidophilus* DDS-1		√	[40]
*Actinobacteria*	√	√	*L. helveticus OFS 1515 and L. fermentum DR9,* *L. casei LC122*	√		[40,41,42]
	[39]	[37,40,41,42]	*L. delbrueckiisubsp. Bulgaricus* 2038, *L. acidophilus* DDS-1, *Lactobacillus paracasei KW3110.* *L. plantarum* TWK10		√	[37,38,39]
Genera:						
*Bacteroides* species diversity	√	√	*L. plantarum* CCFM10, *L. paracasei,* *L. paracasei KW3110*	√		[36,37]
	[39,41]	[36,37]	*L. helveticusOFS 1515 and L. fermentum DR9, L. plantarum* TWK10		√	[39,41]
*Clostridium*	√	√	*L. paracasei KW3110, plantarum* CCFM10, *L. paracasei,* *L. casei LC122*	√		[36,42]
	[36,37]	[42]	*L. paracasei*		√	[37]
*Lactobacillus*	√	√	*L. plantarum* CCFM10, *L. acidophilus* DDS-1, *L. helveticusOFS 1515 and L. fermentum DR9,* *L. casei LC122*	√	√	[36,41,42]
	[36,37,39]	[41,42]				
			*L. paracasei KW3110, L. plantarum* TWK10			[37,39]
Facultative anaerobes:						
*Streptococci*, *Staphylococci*, *Enterococci*, *Enterobacteria*	√	√	*L*. *plantarum* CCFM10, *L. casei LC122*	√		[26,42]
	[37,39]	[36,42]				
			*L. paracasei KW3110, L. plantarum* TWK10		√	[37,39]

## 3. Ageing-Related Decline in Immune System and the Role of *Lactobacillus*

The new medical research suggests that over 90% of human disorders are associated with the immune system. The age-related deteriorations in the immune system (immunosenescence) are increasing very rapidly. Immunosenescence refers to the ageing of immune system. This immune ageing normally leads to higher infection rate, chronic inflammatory diseases, and poor response to vaccination [43,44,45,46]. Mostly, a fundamental change in the immune functioning of Th1/Th2 is associated with growing age that prone the elderly either to self-immune or allergic reactions, which in turn affect other sites of the immune system in later stages [47]. Sharma et al. described age-related changes in immune system of male Swiss albino mice. They observed an imbalance in the Th1/Th2 immune responses and changes in cellular and humoral immunological responses [48]. As such, the mechanism for the reestablishment of age-associated imbalance of Th1/Th2 can be more inspiring to minimize the effects of immunosenescence in aged people. There are some other age-related factors inside the cells, such as interference of redox homeostasis. The free radical theory of ageing properly defines the phenomenon, resulting in oxidative damage to cells and tissues. Therefore, immunosenescence (increased tendency towards diseases) is considered as a big risk for healthy life in older people. Therefore, unsurprisingly the global efforts are expected to minimize the effect of ageing on the immune system and preferably to enhance the redox status in older people. To compete with immunosenescence and age-associated disorders, many studies have been performed, including dietary supplements, vaccination, and anti-inflammatory strategies.

The current methods applied for the treatment of immune disorders still need improvements. The available drugs used for the treatment of different disorders have various side effects, such as stimulation of some components and interruption of immune system. Microbiologically, the gastrointestinal tract is considered as one of the most active environments that contain many bacterial species essential for the maturation of immune cells. Bacteria coming from microbiota and food intake to the intestine flourish together with immune cells associated with lamina propria of the villi. These microbes do not interact with epithelial cells directly but rather help stimulate the maturation and functionality of immune cells via their metabolites [49]. Metagenomic studies have suggested that microbial metabolites have a vital role in host-microbe interactions, including the immune system. Therefore, it is critical to understand the actual mechanism involved in metabolite-mediated host-microbes interaction to develop therapeutic approaches for targeted modulation of the immune system [50]. The consumption of fermented milks and yogurts increased the number of IgA producing cells, macrophages, and specific IgA antibody responses to antigen challenges in contrast with the animals that were not fed with *Lactobacillus* [51]. Another study also showed that taking adequate amounts of lactic acid bacteria as dietary supplements can help the immune system to cope with diseases effectively [52]. In addition, the effects of *Lactobacilli* have also been found to be changing with immune-physiological situations of the study subjects [53]. Therefore, probiotics have been widely investigated for their beneficial effects, particularly their immune-strengthening ability.

Generally, gut permeability increases with ageing. As a result, microbes and microbial contents are released into the blood stream, leading to the activation of the immune system and releasing the pro-inflammatory cytokines. A previous study showed that administration of *Lactobacillus reuteri* 3D8 significantly reduced the levels of primary cytokines, including IL-8, TNF-α, IFN-γ, IL-4, and IGF1 in chickens, which indicates that this strain has the potential to reduce inflammatory responses [54]. Based on the existed literatures, the consumption of probiotics can improve the immune responses of the intestine by (i) boosting the efficiency of dendritic cells, (ii) the stimulation of the activity of natural killer cells, or (iii) stimulation of the secretion of some pro-inflammatory cytokines [55,56]. A typical study showed that administration of a mixture of *L. helveticus* Bar13 and *B. longum* Bar33 enhanced the immune responses in older adults by alleviating naive, activating memory, regulating T cells and B cells, as well as a natural activity while suppressing the T cells compared to placebo [4]. 

Th1 cytokines are a vital group consisting of blood and tissues that prevent different bacterial and viral infections, directing the innate and adaptive immunity to generate cells and antibodies, which play a role against bacteria and viruses. Th2 cytokines can help the body to synthesize IgE and IgA against parasites and mucosal infections. The intragastric administration of the strains *Lactobacillus salivarius* and *L. plantarum* prevented the differentiation of Th2 by releasing IL-10 and TNF-β following Th1 differentiation. After transformation, Th1 cells released cytokines IFN-α1, IFN-β, TNF-α, and IL-12, which inhibited NF-κB signaling pathways and down-regulated the pro-inflammatory cytokines IL-1α and IL-8 [52]. In an in-vivo study, the supplementation of a mixture of *L. plantarum* and Phytochemical epigallocatechin gallate (EGCG) significantly improved the production of Th1/Th2 cytokines and Nrf-2 expression in splenic supernatants and liver, respectively, in aged Swiss albino mice [53]. In addition, studies also indicated that lactic acid bacteria can stimulate the production of cytokines IL-2 and TNF-α, promote Th1 immune responses, and inhibit Th2 immune responses [54,57]. In an experimental model of respiratory allergy, the probiotics maintained a clear balance of Th1, which favored the IgG production rather than IgE immunoglobulin and alleviating the levels of IL-10 and IFN-γ cytokines. During a co-localization study, the authors suggested that Th1 cells are responsible for the release of IFN-γ [58]. Study also showed that *Lactobacillus bulgaricus* N45.10 can modulate the immune responses in mice by preventing Th2 polarization and Th17 cells and increasing Th1 responses through the modulation of STAT6 and T-bet transcription factors [59]. In a chronic asthma mouse model, the administration of *L. rhamnosus* GG reduced the levels of Th2 cytokines and up-regulated Th1 cytokines [58]. *L. plantarum* 06CC2 effectively induced the production of Th1 cytokines activating Th1 immune responses linked to intestinal immunity in normal mice [59]. Some probiotic strains have shown the ex-vivo up-regulation of immune responses of blood mononuclear cells by oral supplementation, suggesting the pro-Th1 immune stimulation. The consumption of three strains of probiotic bacteria for 21 days indicated an increased ex-vivo antitumor activity of natural killer cells in human blood [60]. These evidences suggest that probiotic bacteria can up-regulate certain aspects of systemic level cell mediated immune responses in healthy subjects.

In a DSS-induced colitis model, the oral administration of the strains *Lactobacillus fermentum* KBL374 and KBL375 enhanced innate immune responses by ameliorating the gut barrier function and suppressing the leukocyte infiltration. After the administration of strains, the DSS mice were observed with decreased levels of Th1-, Th2-, and Th17-related cytokines and elevated levels of IL-10 in the colon compared with control. The levels of CD4+CD25+Foxp3+Treg cells were also increased in the mesenteric lymph nodes [61]. Such properties are not attributed to all the genera of *Lactobacilli*, as these characteristics are limited to some strains and depend on the dose administered [62,63]. These probiotic bacteria can increase the number of IgA+ cells in the intestinal mucosal sites and stimulate the macrophages and dendritic cells, which are important for the stimulation of the overall immune system [64]. Some strains can lower the intensity of intestinal infections, which is a clear evidence of highly activated macrophages and phagocytic activity in Peyer’s patches (PPs) [65]. 

**Figure 1:**
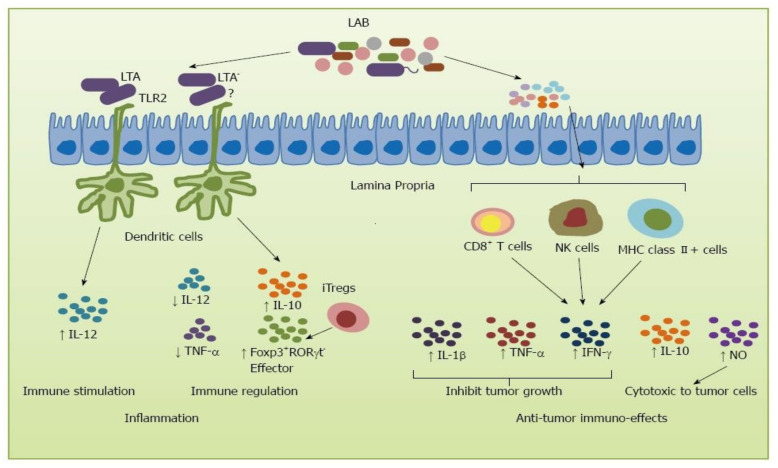
Immune responses induced by lactic acid bacteria. LAB can stimulate immune responses via two major pathways: inflammation and anticancer immune responses. The inflammation pathways include lipoteichoic acid (LTA) which stimulates T cells to release interleukin (IL)-10 and IL-12 and increase effectors Foxp3 + RORγt -Tregs. In the anticancer immune responses pathway, LAB stimulate the immune cells, such as T cells, dendritic cells (DC), natural killer (NK), and MHC class Ⅱ cells to induce IL-10, and tumor necrosis factor (TNF)-α, interferon (IFN)-γ, and IL-1β to inhibit tumor growth. Adopted from [69].

Advancements in the use of probiotics have uncovered the enhanced protection of gut and investigating their effects in distal mucosal sites. It is now evident that probiotics can influence the immune system of the respiratory tract and enhance protection against bacterial and viral infections [66,67]. The microbiota represents a very dense ecosystem that is very complex and is being recognized as an important part of host immune regulation. There is a close connection between the host immune system and intestinal bacteria while their dysregulation can cause inflammatory disorders. Dysbiosis can alter the intestine’s epithelial mucosa, which can initiate inflammation by the up-regulating of cytokine activity [61]. *L. rhamnosus* 1505 can enhance the immune responses against pneumococcal infection [68]. Different strains of *Lactobacillus* have shown promising effects in various disease conditions. They can potentially inhibit the initiation or the progression of diseases through multiple ways. Under pathological conditions, *Lactobacillus* may inhibit diseases by up-regulating the systemic and mucosal immune responses or reducing inflammatory responses to host-microbiota (Figure 1).

On one side, we have the concept that the consumption of functional foods can help in maintaining healthy life by improving health during ageing even though there is a lack of knowledge about the role of *Lactobacilli* as the supplements or the mechanism through which these probiotics improve the intestinal mucosal immunity. However, on other side, there are only few reports showing that probiotic bacteria have a very limited or no specific role in stimulating some aspects of the intestine’s immune response. The actual or predicted significance of probiotic *Lactobacilli* as immunomodulating agents needs further investigation to ensure their actual role, real potential, and mechanism of action.

## 4. Ageing-Related Oxidative Stress and the Role of *Lactobacillus*

Inside the body of an organism, many harmful by-products are produced as a result of aerobic metabolism. For organisms to stay healthy, they need to maintain a low level of these by-products. Reactive oxygen species (ROS) are predominantly formed inside mitochondria [70], which causes damage to lipids, proteins, and even DNA when they are over accumulated inside the cells [71]. Oxidative stress has a great influence on the process of ageing, which is known as the oxidative stress theory of ageing [70]. This oxidative stress also plays a vital role in developing age-associated chronic disorders, like cancer, heart failure, and diabetes. The intra-cellular ROS production is toxic if it is continuously accumulated. The ageing cells have very limited capacity to eliminate these by-products, and if they are exposed to a high toxicity of ROS, the cell functions will be ultimately undermined. In such situations, the body needs an external supply of antioxidants. Among these external antioxidants, several bioactive compounds are available, such as probiotic *Lactobacillus*, which has experimentally proved beneficial for its antioxidant ability.

The strain *L. plantarum* KSFY02 isolated from naturally fermented yogurt can potentially prevent the decline in the indices of different organs caused by oxidative stress in ageing mice. The strain alleviated the activities of different antioxidant enzymes and suppressed the production of nitric oxide and malondialdehyde. Pathological observations showed that the strain can alleviate the liver and spleen damage caused by oxidative ageing [72]. During a study, the supplementation of an exopolysaccharide produced by *L. helveticus* KLDS1.8701 significantly improved the antioxidant status of liver by enhancing the gut microbiota composition in mice [73]. However, the fundamental contribution of ROS in ageing is still contentious. Some studies have revealed that ROS is not the direct source of ageing [74]. Furthermore, ROS has shielding effects in some model organisms, like *Caenorhabditis elegans, Drosophila melanogaster,* and *Saccharomyces cerevisae* [75]. Even so, there is evidence that dietary supplementation by using food products containing bioactive chemicals have strong effects against oxidative stress. After administration of the strain *Lactobacillus delbrueckii subsp. bulgaricus* F17, the activities of GPx in serum and liver and that of superoxide dismutase in serum and brain were significantly increased. At the same time, the levels of malondialdehyde were significantly lowered in liver and serum of ageing mice [76].

In experiments where the system mimics the colon fermentation environment, some strains, like *L. rhamnosus* GG, *L. paracasei* Fn032, and *Lactobacillus spp* 001, have shown promising effects in preventing the production of hydroxyl radicals [77]. Furthermore, the oral supplementation of live recombinant strains of *L. plantarum* has the potential to improve the TNBS-induced colitis through the production of bacterial SOD in rats [78] and mice [79]. Another study showed that *L. helveticus* KLDS1.8701 can potentially improve the liver antioxidative status by modulating the Nrf-2 signaling path way and by attenuating liver oxidative stress through the gut-liver axis [23]. In another study, the cell-free extract of the strain *L. helveticus* CD6 was observed with a higher ability of Fe^2+^ ion chelating [80]. The intracellular content of the strain *L. casei* CRL 431 considerably lowered the aflatoxin-induced lipid peroxidation by alleviating the antioxidant enzyme activity of blood and liver in rats. This study suggested that the intracellular content contains metabolites that can alter the antioxidation system of the host and improve the damage attributed to aflatoxin-induced oxidative stress [81].

The dietary supplementation of *L. fermentum* in pigs caused an increased SOD and GPx in serum, elevated hepatic CAT, and muscle SOD and Cu and Zn-SOD compared to the control group [82]. Additionally, Ahire and his fellows observed that intracellular cell-free extract of *L. helveticus* CD6 producing folate showed antioxidant potential in the same way as the intact cells did [80]. Selenium nanoparticles synthesized by the strain *L. casei* ATCC 393 can prevent the damage caused by oxidative stress in the epithelial barrier function of the intestine by alleviating the ROS-mediated mitochondrial dysfunction through Nrf2 signaling pathway [83]. Interestingly, a previous study found that *L. fermentum* has a whole internal GSH system for the first time [84]. The strains *L.plantarum* AR113 and AR501 enhanced the antioxidant potential of D-galactose-induced ageing mice by alleviating the liver damage caused by oxidative stress and normalizing the activities of different antioxidant enzymes [85]. In case of abnormal gut microbiota, pathogenic bacteria flourish well that induce endotoxins into the blood, which in turn causes considerable oxidative stress. The epithelial ROS generation induced by microbial contact is a much-conserved phenomenon across the phyla. This is a general mechanism through which bacterial communities can influence the redox homeostasis of the host [86].

**Figure 2:**
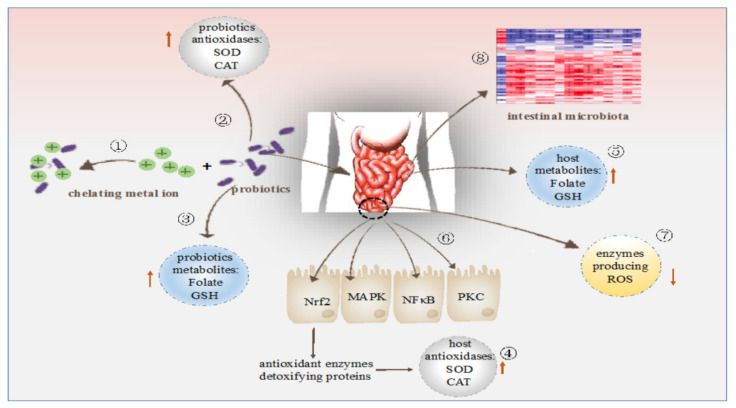
Modulation of antioxidation by probiotics: (1) Chelate metal ions. (2) Possessing their own antioxidation system. (3) Production of antioxidant metabolites. (4) Up-regulation of antioxidant activities of the host. (5) Alleviating the levels of antioxidant metabolites of the host. (6) Regulating signaling pathways. (7) Down-regulation of activities of enzymes producing ROS. (8) Regulation of intestinal microbiota. Adopted from [87].

Therefore, as shown in Figure 2, it is evident that certain strains of *Lactobacillus* can produce metabolites that can prompt the host antioxidant system to cope with oxidative stress. They can also produce certain antioxidant enzymes inside the host body to enhance the host antioxidant system. The administration of cell-free extract and intra-cellular contents of *Lactobacillus* has significantly improved antioxidant enzyme activities of blood and liver, showing that they can influence host antioxidant system through different metabolites [86,87].

## 5. Age-Related Gene Suppression and the Role of *Lactobacillus*

With the onset of ageing, different antioxidant enzymes’ activities are altered, which ultimately leads to the accumulation of reactive oxygen species, causing mitochondrial DNA dysfunction in cells. Ageing suppresses different antioxidant genes, while the expression of pro-oxidant genes is increased. Experimental data suggest that several strains of the genus *Lactobacillus* can potentially produce antioxidant enzymes, such as catalase and superoxide dismutase, protecting against the host ROS. Some *Lactobacillus* strains have been reported to regulate gene expression during ageing and oxidative stress (Table 2). Studies have revealed the mechanism of action of *Lactobacillus* for longevity of life by using the worm *Caenorhabditis elegans. C. elegans* is probably the appropriate and perfect entity for investigating the process of ageing. It is because this worm has an evolutionary safe metabolism and mechanism for host protection, including insulin-like growth factor (IGFs) [88], p38 MAP kinase [89], and transforming growth factor β (TGF-β) signaling pathways [90]. Furthermore, nutritional sources, like bacteria, can play a vital role to prolong the lifetime of *C. elegans* [91]. The ageing process in *C. elegans* is a complex series of changes accompanied by various chemical singling mechanisms. Several genes being controlled in young and elderly in different ways during ageing are thought to be controlled by DAF-16 (fork head box O (FOXO) transcription factor) and SKN-1 (Ortholog of mammalian NF-E2-related factor 2). DAF-16 and SKN-1 have extremely preserved functions to regulate stress tolerance and genes for longevity. Due to the efficient antioxidant effect of *L. rhamnosus* CNCM I 3690, the nematode’s lifetime was expanded due to insulin-like pathway DAF-2/DAF-16 [92]. Feeding with the strain *Lactobacillus gasseri* SBT2055 (LG2055), DAF-2(e1368), and DAF-16 (mgDf50) in mutant *C. elegans* can prolong the lifespan of the worm similarly as it was observed in the case of wild type. On the contrary, the lifespan was not extended by feeding SKN-1 mutant worm with LG2055 [93]. The physiologically controlled performance of SKN-1 may be processed in several ways, including detoxification, immune responses, metabolism, and protection against oxidative stress.

For normal cell functions, it is crucial to maintain low ROS levels. The strain LG2055 can stimulate the host immune system and ROS production. ROS generated byCe-Duox1/BLI-3Ce-Duox1/BLI-3 can activate SKN-1 functions through p38 signaling [94]; however, the NSY-1 and SEK-1 jointly can regulate the p38 MAPK homologue PMK-1, PMK-1 phosphorylates SKN-1, which is transferred to the nuclei of the intestinal cells and activate the expression of phase 2 detoxification genes and respond to oxidative stress [95]. In response to stress and immune regulation, the p38 MAPK is considered to be critical. Papp et al. confirmed that SKN-1 and PMK-1 fundamentally work in immunosenescence [96]. Immunosenescence or immune system malfunction with increasing age is a decisive situation that obstructs healthy ageing [97]. Hence, it is assumed that LG2055 can reduce the age-associated oxidative stress by stimulating the immune system, including p38 MAPK signaling and other pathways. LG2055 slightly extended the lifespan of tir-1 mutant *C. elegans* but did not affect the average lifespan of *nsy-1*, *sek-1*, or *pmk-1* mutant *C. elegans.* In a heat-stressed mice model, the supplementation of Zinc-enriched probiotics significantly enhanced the expression levels of genes SOD1 and SOD2 in various parts of the body. Metallothioneins are proteins that are encoded by a group of zinc-activated transcription genes, which protect cells against the accumulation of ROS. MT-1 and MT-2 are the major proteins involved in ROS scavenging. During the same study, the supplementation of zinc-enriched probiotics significantly increased MT-1 and MT-2 mRNA expression levels [98]. 

**Table 2:** Impact of administration of different *Lactobacillus* strains on gene expression in different organs by using mice model.

Strain	Organ	Gene	Up-Regulated	Down-Regulated	Ref:
*L. mucosae* LMU1001	Intestinal tract	MT1	Yes		[99]
		MT2	Yes		
		GPX1	Yes		
		GPX2	Yes		
		SOD		Yes	
*L. plantarum* CCFM10	Liver	Peroxiredoxin		Yes	[36]
		Glutathione peroxidase		Yes	
		Glutathione reductase		Yes	
		Thioredoxin reductase		Yes	
*L. acidophilus* LaVK2	Liver	PPAR-a	Yes		[100]
		Klotho	Yes		
		SMP-30			
	Kidney	PPAR-a	Yes		
		Klotho	Yes		
		SMP-30		Yes	
*L.plantarum* K68	Liver	TLR4		Yes	[101]
		Foxp3		Yes	
		SOCS3		Yes	
*L.plantarum* AR501	Liver	GST	Yes		[85]
		GCLc	Yes		
		GCLm	Yes		
		NQO1	Yes		
*L.plantarum* CQPC11	Liver	nNOS, eNOS, Cu/Zn-SOD, Mn-SOD, CAT, HO-1, Nrf2, *γ*-GCS, NQO1	Yes		[102]
		iNOS		Yes	
	Spleen	nNOS, eNOS, Cu/Zn-SOD, Mn-SOD, CAT, HO-1, Nrf2, *γ*-GCS, NQO1	Yes		
		iNOS		Yes	

The expression levels of different antioxidant genes were effectively enhanced after the administration of *L. plantarum* KSFY02 in different organs of oxidative stress-induced ageing mice. In contrast, the expression of inducible nitric oxide synthase was down-regulated. During the western blot analysis, the protein expression levels of SOD1, SOD2, CAT, GSH1, and GSH2 were also higher [72]. 

## 6. Brain Ageing and the Role of *Lactobacillus*

Globally, the average lifespan of humans is increasing due to scientific advancements. However, brain disorders, such as neural disorders and neuropsychiatric diseases, still remain a challenge. Anxiety and memory loss are the two most common brain disorders in ageing [103]. Hence, it is necessary to find alternatives that are capable of curing anxiety and memory loss in the aged population. Both inflammation and free radical levels are responsible for increased ageing. Considering their multiple effects, the provision of some probiotic strains may be a suitable choice for the treatment of some important age-associated declines, such as inflammatory disorders [101], oxidative stress [104], and metabolic disorders [105]. Some studies have reported that effects can be shifted from gut to brain region to increase the levels of certain neural monoamines, like dopamine, serotonin, and brain-derived neurotropic factor [BDNF], which are important for normal performance of the brain, like neuronal plasticity and survival [106]. By maintaining the balanced levels of these monoamines, it is believed that the chances of anxiety and memory loss can be minimized up to certain levels [107]. As the levels of these monoamines decrease during ageing, by maintaining their constant levels, the age-associated mental declines can be treated [108].

Recently studies have demonstrated that probiotics can affect the functioning of the central nervous system and its performance through gut-brain axis. *L. helveticus, B. longum,* and *Bifidobacterium breve* have positive effects during anxiety-like behaviors and can strengthen the memory in murine models [109,110]. These improved behaviors were observed with the reestablishment of neural monoamine levels in major brain regions, like the hippocampus and striatum [111]. In fact, only young and middle-aged animals were studied under such experiments. Therefore, the actual effects of probiotics on the age-associated declines in aged population are still contentious. The ageing-associated experiments are expensive and take longer because the animals to be studied need relatively longer time to reproduce. Therefore, in most cases, prematurely aged animals are being studied for age-related research. Senescence-accelerated mouse prone 8 (SAMP8) is a line developed from senescence-resistant mice (SAMR1). It is distinguished by the early onset of ageing, like hair loss, dull hairs, and short lifespan [112]. The SAMP8 mice are similar to the normal mice (SAMR1) for the first four months, but after six months of age, the SAMP8 mice display prominent emotional and memory weakness. Therefore, these disorders are found to happen earlier in SAMP8 mice than the normal mice (SAMR1) [113]. It was confirmed by Rhea and Banks in 2017 that the SAMP8 mice can be used for age-related studies regarding emotion and memory loss. They also stated that the onset of ageing occurs more rapidly in SAMP8 mice after four months of age [114]. 

Along with other factors, ageing also affects the brain both at cellular and functional level, which undermines sensory, cognitive, and motor functions of the brain. In an ageing model, the administration of the strain *L. plantarum* DR7 had several positive effects on the brain of ageing rats, such as enhanced memory, higher cognitive function, and lower anxiety. Analysis of hippocampus showed lower levels of pro-inflammatory cytokines, while the apoptosis biomarker gene was also down-regulated. The expression of neurotransmitter biomarker genes in the hippocampus region showed that the effects of DR7 might be due to the mechanism along the serotonin pathways. All these effects were transferred from gut to brain through gut-brain axis [115].

Different *Lactobacillus* strains have shown beneficial effects on the gut-brain axis. During a human trial, the consumption of the strain *L. plantarum* DR7 by stressed adults for 12 weeks decreased the stress symptoms, anxiety, and total psychological scores when they were compared with the placebo group. Subjects treated with the strain were observed with lowered cortisol levels, pro-inflammatory cytokines (interferon-γ and transforming growth factor-α), and higher anti-inflammatory cytokines in plasma. The strain also enhanced cognitive and memory functions, including basic attention, associate learning, and emotional cognition in adults [116]. An important *Lactobacillus* strain (*L. paracasei* LPPS23) can either alone or in combination with other probiotic bacteria offer considerable effects on the brain and overall nervous system [117,118]. *L. paracasei* NTU 101 can enhance the antioxidative and anti-inflammatory responses [119]. Huang et al. (2018) investigated the effects of LPPS23 on age-related cognitive decline in SAMP8 mice. During the study, they reported that the LPPS23 group exhibited less symptoms of ageing (senescence). In addition, the memory weakening and anxiety-like patterns were lower than the control group. The neural monoamines levels were also lesser in the striatum, hippocampus, and serum of the control group. Furthermore, *L. plantarum* PS23 also accelerated the production of anti-oxidative enzymes like superoxide dismutase (SOD) and glutathione peroxidase (GPx) [120]. Based on the above studies, it can be clearly seen that the great therapeutic potential of using probiotics to treat ageing brain-associated problems. However, more clinical studies are still needed. 

## 7. Skin Ageing and the Role of *Lactobacillus*

Both internal and external factors contribute to skin ageing. Internal factors include genetic alterations monitored by a set of different physiological changes attributed to ageing, including degeneration of epidermal and dermal skin tissue layers and increased dryness [121,122]. External ageing is mostly caused by environmental factors, like UV radiations and toxins like cigarette smoke. The symptoms of external skin ageing are rough wrinkles, decrease in elasticity, epidermal thickness, increased dryness, laxity, coarse appearance, and different pigmentation problems [121]. Most of the age-associated problems appear on the face, neck, forearm, and dorsal regions of hands. As a function of these factors, the ageing process is more evident in these regions. Although both intrinsic and extrinsic skin ageing factors are different, both involve the same molecular mechanism [122,123]. The skin pH is determined by free amino acids, epidermal lactate, and free fatty acids. Usually, skin health is determined by skin pH, but with ageing, the skin pH rises, leading to abnormal conditions. In a double-blind placebo-controlled trial, the ingestion of *L. plantarum* CJLP55 significantly decreased the skin pH. Although the levels of free fatty acids were affected, the total free fatty acids, such as palmitic acids and stearic acids, were lower in the probiotic-treated group [124]. Studies also suggest that Lactobacillus can enhance the protective mechanism in the skin [125]. The dietary supplementation of *L. Johnsonii* displayed inhibition either alone [126] or in combination with carotenoids [127] against early UV-induced skin by regulating the immune cells as well as inflammatory cytokines. 

Further investigations recommend that probiotic supplementations can reduce atopic dermatitis and skin dryness [128,129]. Several experiments by using hairless mice have suggested that along with the control of immune responses in the skin, oral administration of probiotic strains may have anti-ageing effects by suppressing wrinkle formation and increased skin elasticity [130]. As it was reported that *L. plantarum* HY7714 has anti-photoageing properties by reducing the formation of wrinkles and suppressing the epidermal thickness, and after administration of this strain to mice, the skin hydration was increased with the increased level of ceramide by maintaining the serine palmitoyl transferase and the appearance of ceramidase in the skin of mice [131]. In a randomized controlled clinical trial in humans, Lactobacillus strain HY7714 increased skin hydration, inhibited the wrinkle formation, and increased the elasticity of skin gloss [132]. Isoflavones are polyphenolic compounds commonly known as phytoestrogens having antioxidant, anti-tumor, and anti-inflammatory effects. Equol is a major isoflavone. A recent study was conducted to investigate the effect of feeding a fermented product that was fermented with equol producing strain *L. paracasei* JS1 on responses of mice. After analysis of gene expression, it was observed that the messenger RNA of seven skin-related proteins was differentially expressed. The skin moisturizing effects of the strain were also confirmed during the study [133].

## 8. Conclusions

The above-mentioned studies indicate that several age-related physical and physiological aspects of health in laboratory animals and even humans have been improved significantly after the supplementation of *Lactobacillus* strains either alone or in combinations. These aspects include enhancing the ageing-gut microbiota, improvements in host antioxidation system (production of antioxidant enzymes and regulation of gene expression), enhancements of immune system, minimizing the abnormalities related to brain ageing through the gut-brain axis (anxiety and memory loss), and inhibition of both internal and external factors that contribute to skin ageing. Emerging studies at the cellular and molecular level reveal that *Lactobacillus* can increase the resistance of cells, tissues, and organs to ageing and age-related disorders. Although previous studies have shown promising effects regarding the role of *Lactobacillus*, large-scale randomized, placebo-controlled, double-blind clinical trials are still needed to elucidate their substantial role in ageing and age-related problems in humans. The selection of appropriate strain, their optimal dose, method of administration, and duration of treatment period are yet to be confirmed. Progressive research suggests that in the future, probiotic *Lactobacillus* may be used as an alternative treatment to conventional therapy by modulating the gut microbiota and host immune system.
